# Colony-stimulating Factor 3 Receptor Mutated Chronic Neutrophilic Leukemia: A Rare Case Report

**DOI:** 10.7759/cureus.3326

**Published:** 2018-09-18

**Authors:** Bicky Thapa, Christopher Jamhour, Johnny Chahine, Heesun J Rogers, Hamed Daw

**Affiliations:** 1 Internal Medicine, Cleveland Clinic - Fairview Hospital, Cleveland, USA; 2 Biochemistry, University of Miami, Miami, USA; 3 Clinical Pathology, Cleveland Clinic - Fairview Hospital, Cleveland, USA; 4 Hematology and Oncology, Cleveland Clinic - Fairview Hospital, Cleveland, USA

**Keywords:** cnl, leucocytosis, neutrophilia, csf3r, hydroxyurea, ruxolitinib

## Abstract

Chronic neutrophilic leukemia (CNL) is a rare myeloproliferative neoplasm, which is characterized by sustained peripheral leukocytosis with neutrophilia, hepatosplenomegaly, and hypercellularity of the bone marrow, with less than 5% myeloblasts along with normal neutrophil maturation and no dysplasia. In 2016, World Health Organization (WHO) included activating mutations in the gene for colony-stimulating factor 3 receptor (CSF3R) as one of the diagnostic criteria with CSF3RT618I being the most common mutation. We report a rare case of CNL (JAK2V617F negative, BCR-ABL1 negative, CSF3RT618I positive) in an elderly female who had an aggressive clinical course of the disease.

## Introduction

Chronic neutrophilic leukemia (CNL) is a very rare disorder with a dismal prognosis. This disease manifests as leukocytosis with mature granulocytes in peripheral blood and bone marrow [1]. In addition, clinical manifestations that are characteristic of neutrophilic leukemia are hepatosplenomegaly, elevated leukocytic alkaline phosphatase, and elevated serum uric acid. A cytoreductive agent, such as hydroxyurea, is frequently used in patients diagnosed with CNL but most patients show a rapid progression of the disease. This case focuses on the clinical course and management of an elderly woman diagnosed with CNL.

## Case presentation

On routine blood workup, a 79-year-old female was found to have marked leukocytosis. Medical history was significant for coronary artery disease, goiter, carotid artery stenosis, optic neuritis, hyperlipidemia, rheumatic heart disease, type II diabetes mellitus, anemia, and essential hypertension. The physical examination was positive for pallor and splenomegaly.

The initial hematological workup revealed an elevated white blood cell (WBC) count of 74.5 x 10^9^/L (normal range 4.5 – 11 x 10^9^/L), with elevated neutrophils and a monocyte count of 66.61 x 10^9^/L and 8.94 x 10^9^/L, respectively (the normal ranges for neutrophil and monocyte count are 1.45 – 7.50 x 10^9^/L and < 0.87 x 10^9^/L, respectively), the lymphocyte count was 3.23 x 10^9^/L (normal range of 1.00-4.00 x 10^9^/L), hemoglobin 6.4 g/dL (normal 11.5 – 15.5 g/dL), and the platelet count was 234 k/uL (normal range of 150-400 k/uL). The peripheral blood smear showed leukocytosis with absolute neutrophilia and monocytosis with lymphopenia and left-shift. The reverse transcription polymerase chain reaction for BCR-ABL1 was negative. The peripheral blood for the JAK2 V617F mutation was negative and the cytogenetic analysis showed 46, XX normal karyotype.

**Figure 1:**
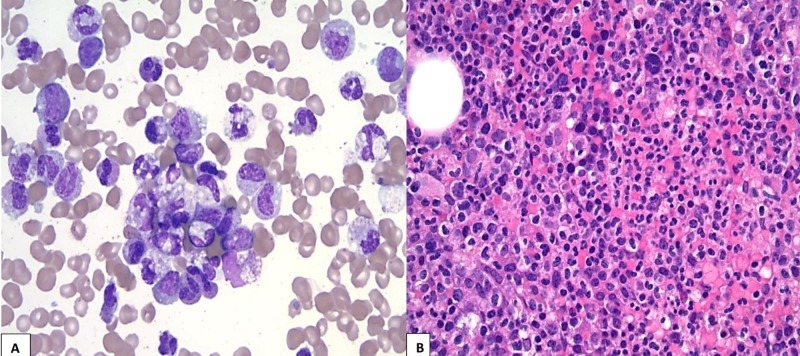
Histology of bone marrow biopsy A: Bone marrow aspirate smear shows granulocytic proliferation, many mature neutrophils with toxic changes, and no increased blasts (Wright-Giemsa stain, x500); B: Bone marrow biopsy shows hypercellularity and marked granulocytic hyperplasia, with increased mature segmented neutrophils (Hematoxylin and Eosin stain, x400)

From the initial evaluation and results from the blood workup, the differential diagnoses include chronic myelogenous leukemia (CML), atypical chronic myeloid leukemia (aCML), chronic myelomonocytic leukemia (CMML), chronic neutrophilic leukemia (CNL), leukemoid reaction, and infections. Further investigation was done with a bone marrow (BM) biopsy, which showed a hypercellular marrow (95%) with marked granulocytic hyperplasia and no increase in the blasts (Figure 1A). The aspirate smear demonstrated granulocytic proliferation, many mature neutrophils, and macrocytic anemia with thrombocytopenia (Figure 1B); it also revealed a low percentage of erythroid precursors (5%) and lymphocytes (2%), respectively, as compared to the normal ranges of 13%-37% for erythroid precursors and 7%-23% for lymphocytes. The genome sequencing was positive for the CSF3RT618I mutation. Our patient fulfilled all the World Health Organization (WHO) diagnostic criteria for CNL with WBC >25 x 10^9^/L and segmented neutrophils >=80%, hypercellular BM, and the presence of the CSF3RT618I mutation. She was managed with hydroxyurea, which is one of the most common cytoreductive agents administered to CNL patients, to control the leukocytosis. The WBC count dropped to 50 x 10^9^/L; however, there was no clinical improvement after six weeks of treatment with hydroxyurea. Clinically, the patient continued to deteriorate and was admitted to the intensive care unit (ICU) as a case of sepsis. During the hospital stay, the patient decided to go for hospice and, unfortunately, expired in the hospital later on.

## Discussion

Chronic neutrophilic leukemia is a rare BCR-ABL1 negative myeloproliferative neoplasm (MPN) characterized by mature granulocytosis without dysgranulopoiesis. Clinically, most patients have an asymptomatic presentation with incidental findings of leukocytosis in blood workup; however, the most common symptom on initial presentation, as per the literature, is fatigue. The other, less common symptoms include night sweats, weight loss, and easy bruising [1]. The clinical course of the CNL can be a chronic phase, an accelerated phase, or a blast phase. Progressive refractory neutrophilia, resistance to the treatment, and transformation to acute myeloid leukemia are potential causes of the clinical progression of the disease in CNL.

The updated WHO classification defined diagnostic criteria, which consist of sustained peripheral leukocytosis with a WBC count of >= 25 x 10^9^/L and >= 80% neutrophils and <10% neutrophil precursors, such as promyelocytes, myelocytes and metamyelocytes; rare myeloblasts, hypercellular bone marrow, and the presence of CSF3RT618I or another activating CSF3R mutation [2].

In 2013, the colony-stimulating factor 3 receptor (CSF3R) gene mutations were discovered in patients diagnosed with CNL and atypical chronic myeloid leukemia (aCML) [3]. This mutation occurs most frequently at the CSF3RT618l position and is commonly identified in CNL patients. Upon further research, it was found that these CSF3R mutations can be found in most CNL patients but not in all aCML patients. A study on mice was done with CSF3RT618l mutated hematopoietic cells transplantation. Subsequently, the subject mice demonstrated a clinicopathological resemblance with CNL, which includes bone marrow hypercellularity and granulocyte infiltrates in the spleen and liver [4].

The JAK2V617F mutation is common in patients with BCR/ABL1 negative MPNs, including CNL, but is rare in CML patients. Besides, most CNL patients with the CSF3R mutation also express other mutations such as the SETBP1 and ASXL1 mutations. As per literature from case series and case reports, patients with concomitant CSF3R and SETBP1 mutations were reported to have a worse prognosis as compared to those without the SETBP1 mutation [5-6].

Since CNL is such a rare disease, no specific treatment exists for these cases. The most common therapy consists of either hydroxyurea, busulfan, or parenteral alfa interferon. These treatment options are effective in improving blood cell counts but there is no evidence to suggest that they have any disease-modifying effect. Treatment with low dose cytarabine, 6-Thioguanine, and leukapheresis have only demonstrated brief responses with transient benefits but no durable response. Allogeneic transplantation can yield beneficial longer-term outcomes when performed in the chronic phase of the CNL. Stem cell transplantation in the blast phase has experienced the worst outcome as per the literature; however, it can be a curative treatment option in some patients. Splenic irradiation and splenectomy are other older modalities of management with the intention of the palliative treatment of symptomatic splenomegaly in CNL. Hydroxyurea is historically the most frequently used cytoreductive agent for patients diagnosed with CNL. Although hydroxyurea has been observed to initially control leukocytosis, eventually, the patients displayed increased levels of transfusion dependence and progression of the disease.

Ruxolitinib is a JAK1/2 kinase inhibitor and is approved by the Food and Drug Administration (FDA) for the treatment of polycythemia vera and cases of intermediate or high-risk myelofibrosis. Targeted therapy with ruxolitinib (currently not FDA approved for CNL) has been reported in the literature for patients with CSF3RT618I mutated CNL [6-8].

## Conclusions

Our case is another addition to the literature; however, the rarity of the disease with an aggressive clinical course is another challenge that requires further research to discover a specific or targeted treatment. Nevertheless, the outcome of an ongoing phase II clinical trial (NCT02092324) in patients with chronic neutrophilic leukemia or atypical chronic myeloid leukemia will help us understand the efficacy and safety of Ruxolitinib.
